# The influence of “Stimix Zoostim” and “Normosil” probiotics on fecal microflora, hematologic indicators, nutrient digestibility, and growth of mother-bonded calves

**DOI:** 10.14202/vetworld.2020.1091-1097

**Published:** 2020-06-15

**Authors:** Fail Khaziakhmetov, Airat Khabirov, Khamit Tagirov, Ruzil Avzalov, Gulnara Tsapalova, Almaz Basharov

**Affiliations:** 1 Department of Physiology, Biochemistry and Feeding Animals, Federal State Budgetary Educational Establishment of Higher Education “Bashkir State Agrarian University,” Ufa, Russian Federation; 2 Department of Technology of Meat, Dairy Products and Chemistry, Federal State Budgetary Educational Establishment of Higher Education “Bashkir State Agrarian University,” Ufa, Russian Federation

**Keywords:** average gain, blood immunoresistance, forage consumption, hematologic indicators, mother-bonded calves, nutrient digestibility

## Abstract

**Aim:**

This paper presents the results of the studies on “Stimix Zoostim” and “Normosil” probiotics and their influence on fecal microflora, hematologic indicators, immunoresistance, nutrient digestibility, and growth intensity of mother-bonded calves.

**Materials and Methods:**

The calves of the control group were fed with their basic diet (BD) without “Stimix Zoostim” or “Normosil,” whereas the calves of the experimental group were fed with their BD supplemented with “Stimix Zoostim” and Normosil.” For 10-20-day-old calves, the daily dose was 10 mL per head, whereas 21-90-day-old calves received 15 mL of probiotics per head per day. The calves of the experimental group were administered probiotics every day. Calves aged 10 to 60 days received probiotics with milk, and then at the age of 61 to 90 days, received probiotics with water. (This is a sequential process. At 60 days old, calf rearing with milk stops, and after that, we use probiotics added to water to rear them). Both groups were administered probiotics twice a day, specifically 50% of the daily dose at each feeding time.

**Results:**

The results in this study proved that “Stimix Zoostim” and “Normosil” probiotics exhibit high probiotic activity and have a positive effect on the calves’ fecal microflora. Feeding the calves with probiotics resulted in a significant increase in the number of normal flora, such as lactic acid bacteria and bifidobacteria, and a decrease in the amount of *Escherichia*. It also resulted in an increase in red blood cells, hemoglobin, and γ-globulins within the physiological range. In our study, we found that the phagocytic reaction in the blood serum of the experimental group was slightly enhanced, suggesting a high response of the body to infectious agents.

**Conclusion:**

Thus, the group of calves receiving probiotic “Stimix Zoostim” exhibited an exceedance of phagocytic activity by 4.8% and the group receiving “Normosil” by 4.4%, in comparison with the control group. The daily dose of 10 mL of probiotics per head for 10-20-day-old calves and 15 mL per head for 21-90-day-old calves also had a positive effect on nutrient digestibility, growth, and forage consumption. The economic benefit per animal was 149.23 and 157.0 rubles, respectively.

## Introduction

In recent years, the scientific and practical interest in probiotics has significantly increased in animal breeding, especially in young stock and poultry breeding, where probiotics are used as disease-preventive and growth-stimulating animal feed additives [1-5].

Probiotics are living microorganisms with a positive effect on the health of young stock, growth performance, immune system, and bowel micropopulation. A better understanding of the mechanisms of the immunomodulatory effects of probiotic bacteria is usually required to give an excellent direction to the development and application of probiotics. Oral administration of probiotic bacteria affects host cytokine levels and thus changes both the natural and adaptive host immune responses. Selected probiotics, including some lactic acid bacillus isolates and enterococcal strains, are considered to prevent *Salmonella* infection. A positive effect of various probiotics on the concentration of hematological blood indicators in calves, such as immunoglobulin G (IgG), was revealed when breeding cattle. For example, increased phagocytic activity in peripheral blood leukocytes was observed when multispecies and multipurpose MMP probiotics, such as *Lactobacillus plantarum*, were added. An increase in total protein and IgG was observed in calves receiving probiotic cultures of *Lactobacillus acidophilus* as well as an increase in erythrocytes and hemoglobin levels. However, there was a reduction of heavy metals, including zinc, by 62.2%, cadmium by 63.7%, and lead by 56.7%, when a probiotic preparation based on soy milk acidified by bifid and propionic acid bacteria was administered to calves, in comparison with the control group [6-8]. After reviewing the literature, we can suggest that probiotic supplements may produce various antibiotic compounds that inhibit the growth of pathogen, prevent gastrointestinal tract diseases, increase digestive enzyme activity, and contribute to antimicrobial protein formation by one strain of *Escherichia coli* to inhibit the growth of its other virulent strains. Some probiotics also had a positive effect on the qualitative characteristics of forage species, including *Lactobacillus animalis* (SB310), *Lactobacillus paracasei* subsp. *paracasei* (SB137), and *Bacillus coagulans* in the ratio of 30:35:35, respectively, as well as “Prolam,” “Batsell,” and other probiotics. The use of these probiotics in the diet of cows and calves contributed to higher intensity of calves’ growth, high milk productivity of cows, and better feed conversion, taking into account new approaches in the preparation of fodder with the use of technical facilities for feeding cattle [9-12]. The range of probiotic use is constantly expanding, and new probiotic additives are entering the market. The sphere of probiotics use is constantly expanding. New probiotic additives with a different set of microorganisms are entering the market. The efficiency of new probiotics to be used in animal breeding requires a broad assessment. The study on the effects of probiotics used in different diets with different nutrient concentrations on animals’ growth and development, nutrient digestibility, blood profiles, fecal microflora, and the release of harmful gases is still a relevant issue. The use of probiotics becomes an integral part of animal breeding because 80% of animal immunity depends on bowel functions, vitamin and amino acid syntheses, food digestion and assimilation of all necessary vitamins and minerals from food, and utilization of metabolic products [13-15].

A new probiotic additive, “Normosil,” is a mixture of living cultures and strains of lactic acid bacteria, such as *Lactobacillus brevis* B-3, *L. plantarum* 8 TIMES, *L. acidophilus* 457, *Enterococcus faecium* UDS 86, and enterosorbents. The following microorganisms comprise the “Stimix Testim” probiotic: *E. coli*, *L. acidophilus*, *Saccharomyces cerevisiae*, *Bacillus subtilis*, *Azotobacter vinelandii*, and *Azotobacter chroococcum*. These are all new microbiological objects in cattle breeding. The influence of these microorganisms on microbiocenosis, ­hematologic blood parameters, immune resistance, nutrient digestibility, and calves’ growth should be closely examined.

This study aimed to investigate the influence of “Normosil” and “Stimix Zoostim” probiotics on fecal microflora, hematologic parameters, immunoresistance, nutrient digestibility, and growth intensity of mother-bonded calves.

## Materials and Methods

### Ethical approval

The study was conducted under the ethical principles approved by the Animal Experiments Ethics Committee, Federal State Budgetary Educational Institution of Higher Professional Education “Bashkir State Agrarian University” (Protocol No. 8 of 28.07.2019).

### Study period and location

Experiments on “Stimix Zoostim” probiotic were conducted at the Bairamgul farm firm in Uchaly, Republic of Bashkortostan. All the studies were conducted from October 1, 2017 until May 2, 2018. A study on “Normosil” was conducted at the Kultaban commercial dairy farm in Baimak, Republic of Bashkortostan. Mother-bonded Holstein black and white calves were the subjects of the experiments that lasted 83-90 days. Comfortable conditions were provided for keeping and feeding the calves. “Normosil” probiotic was provided by the LLC BashInkom Research and Innovation Company, Russia, and “Stimix Zoostim” was obtained from the LLC “Stavropol” Scientific and Production Association (Nevinnomyssk, Russia).

### Experimental group formation and feeding ­conditions for the calves

The calves were grouped using the analog method (gender, date of birth, live weight, etc.), with 10 animals at the age of 7-10 days in each group (50% bull calves and 50% heifer calves). The animal housing and feeding conditions were the same, according to the farm protocol. The calves’ feeding scheme for the experimental period is presented in Table-1.

**Table-1:** Calves feeding scheme for the period of the experiment.

Age, days, decade	Feed, kg/1 head/day				

	Whole milk	Calf starter	Concentrate feed KK-62	Meadow hay	Oat haylage
1-7	5.0	Introduction period	-	-	-
8-30	5.5	0.1	-	Introduction period	-
For the 1^st^ month	161.5	2.3	-	-	-
Fourth 10-day interval	5.0	0.2	0.1	0.3	Introduction period
Fifth 10-day interval	4.0	0.6	0.3	0.4	0.5
Sixth 10-day interval	2.5	0.7	0.6	0.8	1.5
For the 2^nd^ month	115.0	15.0	10.0	15.0	20.0
Seventh 10-day interval	1.35	0.7	0.7	0.9	1.8
Eighth 10-day interval	-	0.6	0.8	0.9	2.2
Ninth 10-day interval	-	0.27	0.9	1.0	3.5
For the 3^rd^ month	13.5	15.7	24	28.0	75.0
Overall in 3 months	290.0	33.0	89.0	43.0	95.0

The calves of the control group were fed with the basic diet (BD) without probiotics, whereas the calves of the experimental group were fed with the BD that included “Stimix Zoostim” and “Normosil” probiotics. For 10-20-day-old calves, the daily dose was 10 mL of probiotics per head, and the 21-90-day-old calves were administered with 15 mL of ­probiotics per head. The calves of this group received probiotics every day. Furthermore, the 10 to 60-day-old calves received probiotics with milk, whereas the 61 to 90-day-old calves received probiotics with water. Both groups received probiotics twice a day, specifically 50% of the daily dose at each feeding time. The calves were fed in accordance with the expected gain and the adopted feeding scheme that was developed based on the actual nutritional food value and consistent with age-food standards. During the ­experiment, the 3-month-old and younger calves were given 290 L of whole milk, 43 kg of meadow hay, 95 kg of vetch and oat mix, 33 kg of pre-starter mix feed, and 89 kg of KK-62 mix feed. The consumption of digestible protein was 127 g/1 energetic feed unit.

### Methods of analysis of morphological properties and calves’ growth

Once a month, all the calves were consistently weighed simultaneously, that is, 2 h before feeding. Blood samples were obtained from the jugular veins on the 90^th^ day, 3 h after the morning feeding. Then, the blood samples were delivered to the laboratory. To determine the level of metabolism and to assess the immune status of experimental calves, blood samples were obtained from four 3-month-old calves from each group before weighing. The blood samples were taken from the jugular vein using Vacutainer tubes. Separate tubes for whole blood and for serum were used for biochemical and immunologic analysis.

The serum was separated from the blood by centrifugation for 10 min at the speed of 2000 r.p.m.

Biochemical analysis of blood serum was conducted using a semiautomatic Stat Fax analyzer. An automatic hematology analyzer, Abacus Junior B, together with “Olveks” test kits, was used to obtain hematologic parameters. Each analyzed indicator was tested separately according to the approved analyzer methods.

The phagocytic activity of neutrophils was determined using inert particles of polystyrene latex (Sigma-Aldrich, USA), with a diameter of 0.80 microns dissolved in Hanks’ solution to a concentration ratio of 1:10.

Immunoglobulin A, G, and M levels were determined using the Mancini radial diffusion method. Immune serum against antibodies (Abs) of a particular class (IgG, IgM, or IgA) was introduced into a melted agar solution. After the melted agar solution was frozen, the Abs were evenly distributed on it. The material (antigen [Ag]) introduced into the basin diffused radially into the gel column. In the area of equivalence in the Ag-Ab reaction (instead of ­precipitin bands), precipitin rings were formed around the Ag basin since the concentration of Ab is the same everywhere. The diameter of the precipitin ring is directly proportional to the Ag concentration in the test fluid.

Quantitation of circulating immune complexes

The blood samples obtained from the calves’ jugular vein were kept for 30 min at room temperature. The obtained blood serum was centrifuged for 10-15 min at a speed of 400 r.p.m. This method was based on the selective precipitation of the Ag-Ab complexes in 3.75% solution of polyethylene glycol M-6000, with the subsequent photometric determination of the precipitate density at 450 nm.

### Microbiological examination of feces

Microbiological examination of calves’ feces was performed on the 90^th^ day of the experiment to determine the microflora composition and microorganism classification (lacto- and bifidobacteria or *Escherichia*). The researchers, using sterile rubber gloves, collected the fecal samples from the rectum and placed them into sterile plastic tubes. Before the analysis for coliform bacterium, lactobacilli, and bifidobacteria, the samples were stored in a freezer at −20°C.

### Microbiocenosis of calves’ feces

Microbiocenosis of calves’ feces involves the counting and classification of microorganisms in calves’ feces. The researchers, using sterile rubber gloves, collected the fecal samples from the rectum and placed them into sterile plastic tubes.

For a comparative assessment of intestinal microbiocenosis, depending on the clinical condition of calves, a series of bacteriological analyses of the intestinal contents of clinically healthy newborn calves (n=12) were conducted. Microbiological studies were conducted using standard methods.

To isolate aerobic and facultative anaerobic microflora from the feces, a series of ten-fold dilutions were prepared, which are sown on dense nutrient environments. The crops were incubated in a thermostat at a temperature of 37°C for 24-48 h. The morphological properties of isolated cultures of microorganisms were studied using light microscopy.

For life-time bacteriological diagnostics of intestinal disorders, the feces of calves that were not treated with antibacterial drugs were sent to the laboratory. Here, 2-3 g of fecal samples were obtained directly from the rectum of 5-6 sick animals from one farm using a boiled rubber catheter. Then, the samples were placed in sterile test tubes. The labeled tubes were packed into a plastic bag or cartons. If it was impossible to quickly deliver the fecal samples to the laboratory (3-4 h after taking them), they were preserved with 30% sterile glycerine solution at a ratio of 1:2.

The level of *E. coli* was calculated using differential environments of Endo, Levin, or MacConkey agars and an environment with sorbitol. The crops were incubated at 37°C for 18-24 h. At the end of the sowing period, the amount of *E. coli* in dark red colonies with a metallic luster was calculated.

### Statistical analysis

Statistical analysis was conducted using the Statistica 10 program (StartSoft Co.). Quantitative data are expressed as an arithmetic mean and its standard error (X±Sx). The reliability of differences among groups was assessed using Student’s t-test, and the differences in the compared groups that had p<0.05 were considered to be statistically significant.

## Results

Microbiocenosis of feces of 3 month-old calves, mln COG/g (Χ±Sx, n=3) are presented in Table-2. This study has shown that the inclusion of probiotics “Stimix Zoostim” and “Normosil” in the diet of calves contributed to the increase in the number of lacto- and bifidobacteria by 27.0%-37.6% and 35.7%-17.6%, respectively, whereas *Escherichia* reduced by 31.3%-21.3%, compared with the control group (p<0.05).

**Table-2:** Microbiocenosis of feces of 3-month-old calves, mln. UFC/g (X±Sx, n=3).

Indicator	Probiotic “Stimix Zoostim”		Probiotic “Normosil”	
	
	Control group 1	Experimental group 2	Control group 1	Experimental group 2
*Lactobacillus*	5.48±0.36	6.96±0.32*	8.01±0.16	10.87±0.92*
Bifid bacteria	6.12±0.38	8.42±0.44*	9.07±0.35	10.67±0.41*
*Escherichia*	5.12±0.42	3.52±0.36*	6.40±0.42	5.04±0.22*

* The difference is significant at p<0.05 with respect to control group 1

The morphological composition and biochemical blood indicators of the 3-month-old calves of the experimental group are presented in Table-3.

**Table-3:** Morphological structure and biochemical blood indicators of 3-month-old calves (X±Sx, n=3).

Indicator	Probiotic “Stimix Zoostim”		Probiotic “Normosil”	
	
	Control group 1	Experimental group 2	Control group 1	Experimental group 2
Leukocytes (WBC), 10^9^/l	9.3±0.74	10.4±0.82	9.76±0.36	9.53±0.48
Red blood cells (RBC), 10^12^/l	7.5±0.16	8.2±0.19*	7.83±0.03	7.96±0.04*
Hemoglobin (Hb), g/l	102.6±2.52	114.2±3.12*	114.01±2.83	120.32±2.03*
Total protein, g/l	62.4±3.12	65.8±3.18	63.15±0.89	65.88±0.71*
Protein fractions, %				
Albumins	41.8±2.26	38.8±1.82	42.8±2.32	40.6±2.48
a-globulins	14.8±1.14	11.1±0.73	15.6±1.28	12.1±1.82
β-globulins	14.6±1.82	11.9±0.78	15.4±1.64	12.8±2.88
γ-globulins	28.8±2.22	38.2±2.26*	26.2±2.02	34.5±2.18*
Glucose, mmol/l	3.2±0.18	3.4±0.22	3.45±0.51	3.41±0.36
Aspartate transaminase, mmol/(h*l)	0.66±0.06	0.68±0.12	0.78±0.09	0.76±0.22
Alanine aminotransferase, mmol/(h*l)	0.38±0.18	0.42±0.16	0.42±0.19	0.46±0.18
Total calcium, mmol/l	2.6±0.14	2.8±0.22	2.77±0.03	2.93±0.06*
Inorganic phosphorus, mmol/l	1.4±0.08	1.6±0.12	1.93±0.03	1.94±0.05

* The difference is significant at p<0.05 with respect to control group 1

Within the physiological norm, the use of “Stimix Zoostim” probiotic contributed to the increase in red blood cells by 9.3%, hemoglobin level by 11.3%, and γ-globulins by 32.6%, compared with the control group (p<0.05). The use of “Normosil” probiotic also gave positive results. Thus, an increase in red blood cells by 1.7%, hemoglobin level by 5.5%, total protein by 4.3%, γ-globulins by 31.7%, and total calcium by 5.8% was noted, compared with the control group (p<0.05). Such changes in blood composition indicate high natural resistance typical for intensively growing animals.

The results obtained from the serum examination for the calves’ body resistance elucidated the immunostimulatory effect of the studied probiotics on the non-specific and humoral immunity factors (Table-4).

**Table-4:** Immunoresistance indicators of calves’ blood (±Sx, n=3).

Indicator	Probiotic “Stimix Zoostim”		Probiotic “Normosil”	
	
	Control group 1	Experimental group 2	Control group 1	Experimental group 2
Phagocytic activity, %	71.4±1.12	76.2±0.86*	72.8±1.24	77.2±0.98*
Ig A, mg/ml	3.9±0.42	4.8±0.38	4.08±0.66	4.92±0.48
Ig M, mg/ml	3.2±0.42	3.7±0.32	3.4±0.62	3.6±0.42
Ig G, mg/ml	20.6±1.18	18.6±1.18	20.8±1.22	19.8±1.28
Total Ig E, IU/ml	34.8±4.26	58.0±4.22*	36.8±3.16	48.0±3.26
Circulating immune complexes, ea.	62.8±1.22	64.8±1.28	62.9±1.22	66.6±1.18

* The difference is significant at p<0.05 with respect to control group 1

During the study, we have found that the phagocytic reaction in the blood serum of the experimental calves was slightly enhanced, which indicated the calves’ strong resistance to infectious agents. The group of calves receiving “Stimix Zoostim” exhibited an exceedance of phagocytic activity by 4.8% and the group receiving “Normosil” by 4.4%, in comparison with the control group. Moreover, a significant increase in IgE by 66.7% in calves receiving “Stimix Zoostim” was also noted, in comparison with the control group.

An increase in protein digestibility in calves receiving “Stimix Zoostim” and “Normosil” was also observed during the research. The protein digestibility coefficients were 84.9% (p<0.05) and 85.2% (p<0.01), respectively, which are 3.8%-3.1% higher than that of the control group (Table-5).

**Table-5:** The results of calves breeding when using probiotics “Stimix Zoostim” and “Normosil” (X±Sx, n=10).

Indicator	Probiotic “Stimix Zoostim”		Probiotic “Normosil”	
	
	Control group 1	Experimental group 2	Control group 1	Experimental group 2
Body weight at the beginning of the experiment, kg	34.7±0.73	34.6±0.62	40.4±1.56	40.0±0.90
Body weight at the end of the experiment, kg	95.1±1.55	100.4±1.87*	95.4±1.03	99.4±1.29*
Absolute gain, kg	60.4±1.24	65.8±1.96*	55.0±1.31	59.4±1.34*
Average daily growth, g	728.0±14.92	793.0±23.66*	611.1±9.55	660.0±10.98**
To be controlled, %	100	108.9	100	107.4
Energetic feed unit consumption per 1 kg of the body weight gain	4.7	4.32	3.90	3.61
To be controlled, %	-	91.9	100	92.6
Safety of livestock, %	100	100	100	100
Economic effect per an animal, RUB	-	149.23	-	157.0

* The difference is significant at p<0.05;

** <0.01 with respect to control group 1

With 100% livability of calves from the experimental group, the average daily increase was 8.9%-7.4% higher, whereas feed costs were reduced per 1 kg of the body weight by 8.1%-7.4% compared with the control group. The results of the effective dose testing for both probiotics revealed that the economic effect per animal was 149.23 and 157.0 rubles, respectively.

## Discussion

Probiotics are an alternative to antibiotics, but they are not addictive substances. Grønvold *et al*. [16], in their studies, revealed that after 5 days of penicillin treatment, the antimicrobial sensitivity of *E. coli* increased in calves.

Bacterial enzymes, including cellulose-decomposing ones, improve the conversion of coarse feed and the absorption of phytin. The increase in the number of rumen microorganism stimulates the reproduction of protists that use bacteria as a food source. The protists’ enzymes play a significant role in the digestion process. In addition, probiotics reduce the presence of pathogenic and opportunistic microorganisms in the digestive tract of animals, creating conditions for improving the environment and preventing young animals from getting sick.

The efficiency of probiotics depends on different types of bacteria. As such, the latters are used for the production of different types of probiotics. Probiotic microorganisms multiply in the intestines of animals and secrete various biologically active substances, under the influence of which the digestive processes are activated. This leads to an increase in body weight gain and contributes to both the safety of livestock and the efficiency of growth of young animals.Klein-Jöbstl *et al*. [17] studied the fecal microbiota of the Simmental breed calves to assess the dynamics of the microbial community depending on the age. As part of the study, taxonomic units were assigned to 296 genera and 17 types, with *Bacteroidetes*, *Firmicutes*, and *Proteobacteria* being the most common among them.

The research results of Oikonomou *et al*. [18] revealed that the microbial fecal diversity is closely related to the age, immunity, and growth rate of a calf. The results of our studies proved a definite effect of “Normosil” and “Stimix Zoostim” probiotics on the calves’ microbiocenosis.

When calves receive probiotics, the number of lactobacilli and bifidobacteria in their bodies significantly increases, whereas the amount of *E. coli* decreases. Our findings are consistent with those of Roodposhti *et al*. [19] and the data of Dar *et al*. [20], which confirm that the number of *E. coli* in calves that received probiotic, prebiotic, and synbiotic treatments decreased on the 56^th^ day (p<0.05).

The changes in biochemical blood serum parameters were within the normal range. No significant difference was observed. Similar data were presented in the studies of Dar *et al*. [21], showing that there was no significant effect of probiotic, prebiotic, and synbiotic treatments on glucose, aspartate transaminase, and alanine aminotransferase levels.

According to Al-Saiady [7], calf groups receiving probiotics exhibited a significant increase in serum IgG concentration. Their bodyweight also increased significantly throughout the experimental period.

The increase in hemoglobin level was also confirmed by Dar *et al*. [22]. We assessed the influence of probiotics, prebiotics, and synbiotics on the hematological parameters of the calves and found that from the 30^th^ day of the experiment until the end of the research, the calves’ hemoglobin levels were increasing.

In our study, it was found that the phagocytic reaction in the blood serum of the experimental group was slightly enhanced, which meant a high response of the body to infectious agents. Thus, the group of calves receiving “Stimix Zoostim” revealed an exceedance of the phagocytic activity by 4.8% and the group receiving “Normosil” by 4.4%, in comparison with the control group. The data obtained are consistent with the results of Indart [4]. According to his study, the use of probiotics in calf breeding led to a significant increase in the phagocytic activity of peripheral blood leukocytes.

Probiotic supplementation at a dose of 10 mL/day/10-20-day-old calves, or 15 mL/day/21-90-day-old calves, had a positive effect on nutrient digestibility. The works of Hossain [23] contain similar data, according to which the digestibility coefficient of organic matter, crude protein, and crude fiber was also increased (p<0.05).

The growth intensity of calves receiving probiotics was increased. Similar results were presented in the works of many scientists. Jatkauskas and Vrotniakiene [24] had shown in their study that when calves received Optisaf probiotic at a dose of 10 g/animal/day, the bodyweight of 6-month-old calves increased by 5.03% (p<0.01), in comparison with the control group.

According to Kocyigit *et al*. [25], the use of microbial preparations led to a weight increase in calves, although the weight gain and feed efficiency coefficients were not significant. However, the data obtained by Bakhshi *et al*. [26] proved that some probiotics were not effective and did not have an influence on the calves’ growth. Riddell *et al*. [27] attempted to estimate the effects of a *Bacillus*-based probiotic together with milk substitute and starter on calf growth. As a result, there was no difference in the growth and health of the calves in the control and experimental groups.

The use of “Stimix Zoostim” and “Normosil” probiotics helped in the reduction of feed consumption per 1 kg of body weight gain. The data we have obtained are consistent with the results of the study conducted by Ülger [28], who found that calves receiving probiotics consumed 2% more feed than the calves from the control group. The feed conversion rate was 9.52% better than that of the control group (p<0.05).

## Conclusion

The results of this study proved that “Stimix Zoostim” and “Normosil” exhibit high probiotic activity and have a positive effect on the calves’ fecal microflora. Feeding the calves with probiotics resulted in a significant increase in the number of normal flora bacteria, such as lactic acid bacteria and bifidobacteria; however, the amount of *Escherichia* decreased. Red blood cells, hemoglobin level, and γ-globulins increased within the normal physiological range. During our study, the phagocytic reaction in the blood serum of the experimental group was found to be slightly enhanced, which meant a high response to infectious agents. Thus, the group of calves receiving “Stimix Zoostim” exhibited an exceedance of phagocytic activity by 4.8% and the group receiving “Normosil” by 4.4%, in comparison with the control group. If 10-20-day-old calves received 10 mL of probiotics per day, or 21-90-day-old calves received 15 mL/day, then there will be a positive effect on nutrient digestibility, growth intensity, and forage consumption. Based on our study, the economic benefit per animal was 149.23 and 157.0 rubles, respectively.

## Authors’ Contributions

FK: Coordination of the experiments and generalization of results of scientific and economic experiments, primary author, and general manager, drafted and revised the manuscript. AK: Review of literature on the use of Normosil probiotic. KT: Review of literature on the use of Stimix Zoostim probiotic; RA: Generalization of the literature review and organization of discussion of the results of scientific and economic experiments. GT: Biometric processing of digital material. AB: Biometric processing of digital material and preparation of the manuscript. All authors read and approved the final manuscript.
